# Nutrition knowledge awareness and associating factors among school-age children in Chongqing: a 2023–2024 survey

**DOI:** 10.3389/fnut.2026.1935221

**Published:** 2026-10-19

**Authors:** Miaoran Wang, Dong Wang, Fang Li, Jiao Huo, Ping Feng, Jingrong Chen

**Affiliations:** 1 Chongqing Center for Disease Control and Prevention, Chongqing, China; 2 Chongqing Jiangbei District Center for Disease Control and Prevention, Chongqing, China

**Keywords:** associating factors, health education, health knowledge, nutrition knowledge, school-age children

## Abstract

**Objective:**

To assess the nutritional knowledge level and its Associating Factors among school-aged children in Chongqing (2023–2024).

**Methods:**

A stratified random cluster sampling method was used to survey 4,980 children (aged 6–17) across 8 districts. The questionnaire covered five dimensions: food selection, safety, classification, dietary recommendations, and growth. Participants scoring ≥75/100 were classified as knowledgeable. Logistic regression analyzed associating factor.

**Results:**

The overall awareness rate was 48.51%. Specifically, the rates were 80.56% for lower-grade primary school students, 51.39% for upper-grade primary school students, 38.30% for junior high school students, and 22.42% for senior high school students. The awareness rate of nutrition and health knowledge, as well as food safety dimension knowledge, among lower- and upper-grade primary school students, the awareness rate was higher in girls than in boys. The number of knowledge gaps (items with <25% accuracy) was 0, 3, 7, and 8 for lower-grade primary, upper-grade primary, junior high, and senior high school students, respectively. Logistic regression analysis indicated that gender, school meals, boarding status, and awareness of parents’ education and employment type were associated with the level of nutrition and health knowledge among children across different educational stages.

**Conclusion:**

Nutritional knowledge among school-aged children in Chongqing requires substantial improvement. Precision health education targeting specific knowledge gaps and vulnerable populations is urgently needed to comprehensively enhance national nutritional literacy.

## 1 Introduction

Children and adolescents are in a critical period of growth and development, as well as a crucial stage for the formation of dietary behaviors and health concepts (1). Adequate nutritional intake has profound implications for their cognitive development, physical growth, and long-term health (2, 3). However, with rapid socioeconomic transitions and lifestyle changes, children and adolescents in China face multiple challenges, including the dual burden of malnutrition and overnutrition (4–6), unbalanced dietary patterns (7), and insufficient physical activity (8). Consequently, chronic diseases such as obesity, hypertension, and diabetes are increasingly occurring at younger ages (9, 10). Numerous studies have demonstrated that nutritional knowledge is closely associated with individual dietary behaviors, serving as a fundamental prerequisite for promoting behavioral change (11, 12). Therefore, systematically assessing the nutritional knowledge level of children and adolescents, identifying knowledge gaps and their determinants, and formulating targeted educational strategies are of great practical significance. Enhancing national nutritional literacy is one of the core objectives of the “Healthy China” initiative. Multiple national policy documents, including the *Healthy China 2030 Planning Outline* and the *National Nutrition Plan (2017–2030)*, have explicitly proposed increasing public awareness of nutritional knowledge and integrating nutrition education into school curricula. Against this backdrop, reviewing the current status of nutritional knowledge among children and adolescents, analyzing its influencing factors and core deficiencies, holds substantial practical value for developing precise and systematic nutritional intervention strategies.

## 2 Subjects and methods

### 2.1 Study subjects

Data were obtained from the Chongqing survey site of the 2023–2024 National Survey on Nutritional Knowledge Awareness among School-Aged Children in China (13). A stratified random cluster sampling method was employed to select six urban districts (Yuzhong, Nan’an, Jiangbei, Qijiang, Tongliang, and Hechuan) and two rural counties (Fengjie and Xiushan) in Chongqing. Within each survey site, two primary schools, one junior high school, and one senior high school (including vocational high schools, secondary specialized schools, and technical schools) were randomly selected. Stratification was conducted by grade (one-year intervals, resulting in 12 age groups), and random cluster sampling was performed at the classroom level to construct the study sample. This study surveyed a total of 4,984 students aged 6–17 years, including those in lower primary school (grades 1–3), upper primary school (grades 4–6), junior high school, and senior high school. Following data cleaning, a total of 4,980 valid samples were included in the final analysis.

This study was approved by the Ethics Committee of the National Institute for Nutrition and Health, Chinese Center for Disease Control and Prevention (Approval No. 2022–037). Written informed consent was obtained from all participants or their parents/guardians.

### 2.2 Methods

A self-administered questionnaire was used to assess the participants’ nutritional knowledge and related characteristics. The survey instrument utilized was the Nutritional Knowledge Questionnaire for Chinese School-Aged Children, which had been validated by experts and tested for reliability and validity. The questionnaire was adapted into four versions tailored to different educational stages: lower primary school, upper primary school, junior high school, and senior high school. The number of items in each version was 25, 24, 26, and 25, respectively. The *Cronbach’s α* coefficients were 0.54, 0.76, 0.79, and 0.79, respectively, indicating acceptable to good reliability for all versions except the lower primary school version. In the confirmatory factor analysis, the chi-square to degrees of freedom ratio (χ^2^/df) was 1.49, 1.30, 1.66, and 1.51, respectively, all below the recommended threshold of 3. Additionally, the root mean square error of approximation (RMSEA) was 0.04, 0.04, 0.05, and 0.04, respectively, all ≤ 0.05. These results indicate that the dimensional structure of the questionnaire aligns well with the theoretical framework, demonstrating good construct validity (14).

### 2.3 Survey content

The questionnaire comprised two main sections: demographic characteristics and nutritional knowledge. The demographic section collected information on gender, age, school attended, boarding status, parental background, and family circumstances. The nutritional knowledge section was structured across five dimensions: dietary recommendations, food classification and characteristics, the relationship between food and growth and development as well as health, food selection, and food safety.

### 2.4 Scoring criteria

The nutritional knowledge questionnaire had a maximum score of 100 points. A total score of ≥ 75 was defined as having adequate nutritional knowledge awareness. Similarly, awareness for each individual dimension was defined as achieving a score of ≥ 75% of the maximum possible score for that dimension. For scoring purposes, each single-choice question was treated as one knowledge point, while each option in a multiple-choice question was considered an independent knowledge point. The correct response rate was calculated as the proportion of participants who correctly answered a specific question or selected the correct option(s) out of the total study population.

### 2.5 Quality control

Prior to the survey, a standardized protocol and operational manual were used to train the personnel in charge at each survey site, who then conducted secondary training at their respective sites, ensuring that participants were selected strictly in accordance with the specified methods. During the field investigation, trained professionals explained the purpose of the survey and provided guidance on completing the questionnaires. In cases where respondents had limited language comprehension or language barriers, interviewers were permitted to provide explanations based on the original intent, without employing leading or suggestive language. On-site quality control personnel reviewed and verified 15 questionnaires each day; any omissions or logical inconsistencies identified were promptly addressed by having respondents complete the missing information. During data analysis, the data underwent cleaning and logical verification, and unqualified questionnaires were excluded.

### 2.6 Statistical analysis

Statistical analyses were performed using SPSS 25.0 software. Continuous variables, specifically nutrition and health knowledge scores, were described as $\bar{x}\pm s$ , while categorical variables were presented as frequencies and proportions. For the complex sampling analysis, urban/rural residence and grade were designated as stratification variables, and class as the cluster variable. Sampling weights were set to ‘1’ for all participants due to the unavailability of post-stratification data. Subsequently, a complex samples chi-square test was utilized to compare the differences in awareness rates among different groups, and a complex samples binary logistic regression was applied to identify the determinants of nutrition and health knowledge awareness. The significance level was set at *α =* 0.05. If the overall difference was statistically significant, pairwise comparisons were conducted using the *Bonferroni* method to adjust the significance level.

## 3 Results

### 3.1 General characteristics

A total of 4,980 students were surveyed, including 2,467 males (49.54%) and 2,513 females (50.46%). The scores and awareness rates of nutritional health knowledge across different educational stages and dimensions are shown in Table 1.

**Table 1:** Dimension-specific nutrition and health knowledge scores and awareness rates by educational level.

Dimension	Lower grades of primary school		Upper grades of primary school		Junior high school		Senior high school		Total	
	Score ( $\bar{x}\pm s$ )	Awareness rate *N* (*r%*)	Score ( $\bar{x}\pm s$ )	Awareness rate *N* (*r%*)	Score ( $\bar{x}\pm s$ )	Awareness rate *N* (r%)	Score ( $\bar{x}\pm s$ )	Awareness rate *N* (r%)	Score ( $\bar{x}\pm s$ )	Awareness rate *N* (r%)
Dietary recommendations	28.05 ± 6.20	773 (59.88%)	18.11 ± 4.89	423 (33.71%)	16.83 ± 4.29	214 (18.34%)	14.61 ± 4.47	99 (7.81%)	19.49 ± 7.25	1,509 (30.30%)
Food classification and characteristics	13.57 ± 2.66	970 (75.14%)	15.65 ± 4.11	577 (45.98%)	13.23 ± 3.78	524 (44.90%)	9.51 ± 3.32	403 (31.81%)	12.98 ± 4.15	2,474 (49.68%)
Food in relation to growth and health	17.61 ± 3.77	1,153 (89.31%)	12.10 ± 2.62	823 (65.58%)	12.04 ± 2.94	770 (65.98%)	14.03 ± 3.61	667 (52.64%)	14.00 ± 3.99	3,413 (68.53%)
Food choices	11.99 ± 3.94	721 (55.85%)	12.80 ± 3.25	624 (49.72%)	15.48 ± 3.36	799 (68.47%)	14.45 ± 3.31	707 (55.80%)	13.64 ± 3.74	2,851 (57.25%)
Food safety	12.41 ± 3.65	780 (60.42%)	12.86 ± 3.05	921 (73.39%)	11.73 ± 2.69	772 (66.15%)	11.91 ± 2.87	872 (68.82%)	12.23 ± 3.12	3,345 (67.17%)
Nutrition and health knowledge	83.63 ± 13.11	1,040 (80.56%)	72.33 ± 13.59	645 (51.39%)	69.31 ± 12.90	447 (38.30%)	64.51 ± 13.76	284 (22.42%)	72.56 ± 15.13	2,416 (48.51%)

### 3.2 Differences in knowledge dimensions across educational stages

Statistically significant differences were observed across educational stages in students’ overall nutrition and health knowledge, as well as in their awareness rates and scores across various knowledge dimensions (*p* < 0.05). In the dimensions of dietary recommendations and food in relation to growth and health, the awareness rates and scores of lower-grade primary school students were significantly higher than those of students in other stages (*p* < 0.05). In the food classification and characteristics dimension, the awareness rates and scores of senior high school students were notably lower than those of their counterparts in other stages (*p* < 0.05). Regarding the food choices dimension, junior high school students demonstrated significantly higher awareness rates and scores than students in other stages (*p* < 0.05). In the dimension of food safety, the awareness rates and scores of junior and senior high school students were significantly lower than those of elementary school students (*p* < 0.05) (Figure 1).

**Figure 1:**
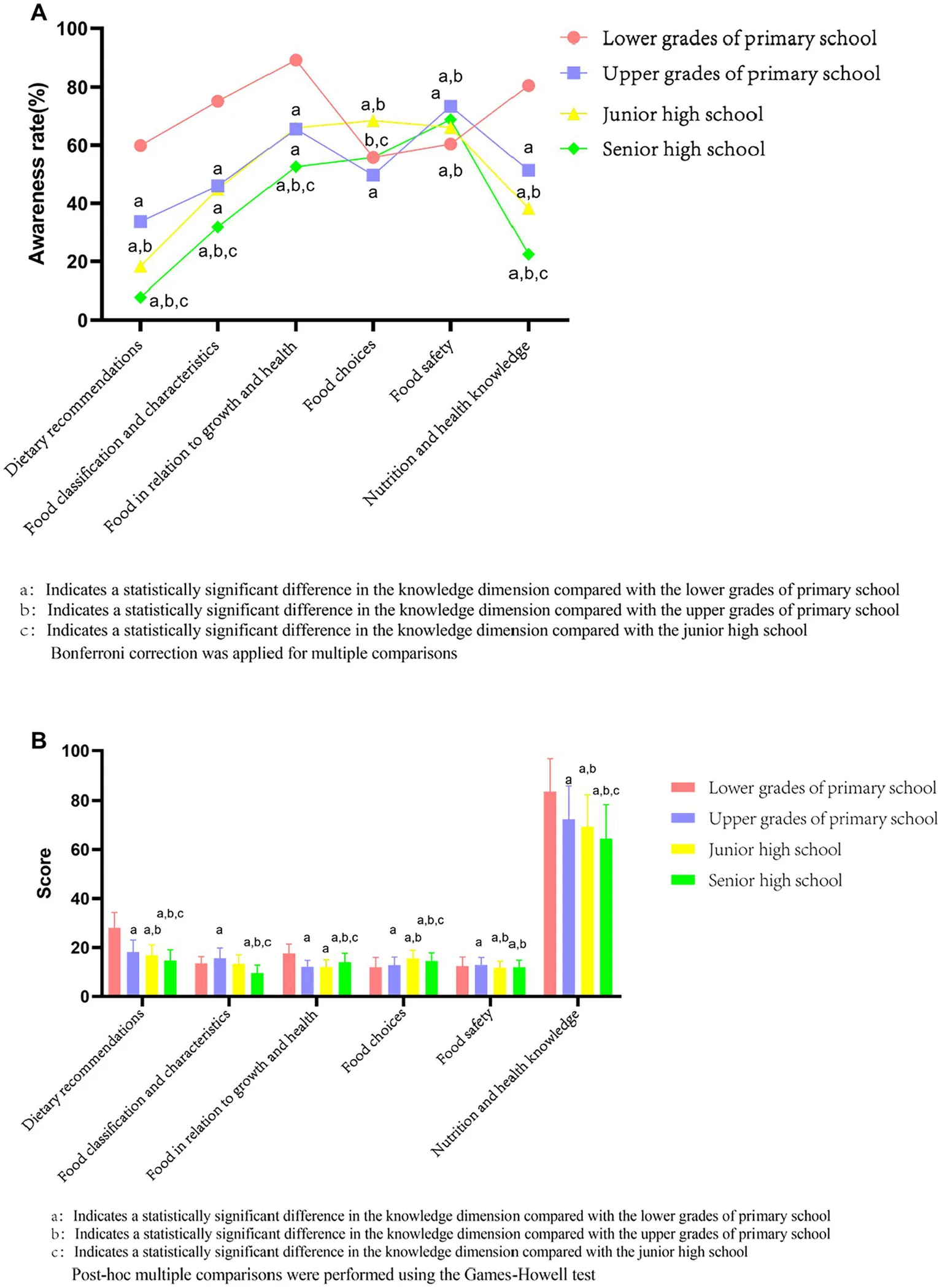
The difference in the awareness rate **(A)** and score **(B)** of the knowledge dimension among school-age children of different school stages.

### 3.3 Gender differences in knowledge dimensions

Among lower- and upper-grade primary school students, girls had significantly higher awareness rates and scores of nutrition and health knowledge than boys (*P* < 0.05). In the dietary recommendations module, girls in upper-grade primary school had higher awareness rates and scores than boys (*p* < 0.05). In the food choices module, girls in lower-grade primary school had higher awareness rates and scores than boys (*P* < 0.05). In the food safety module, girls across all four educational stages had higher awareness rates and scores than boys (*P* < 0.05) (Figure 2).

**Figure 2:**
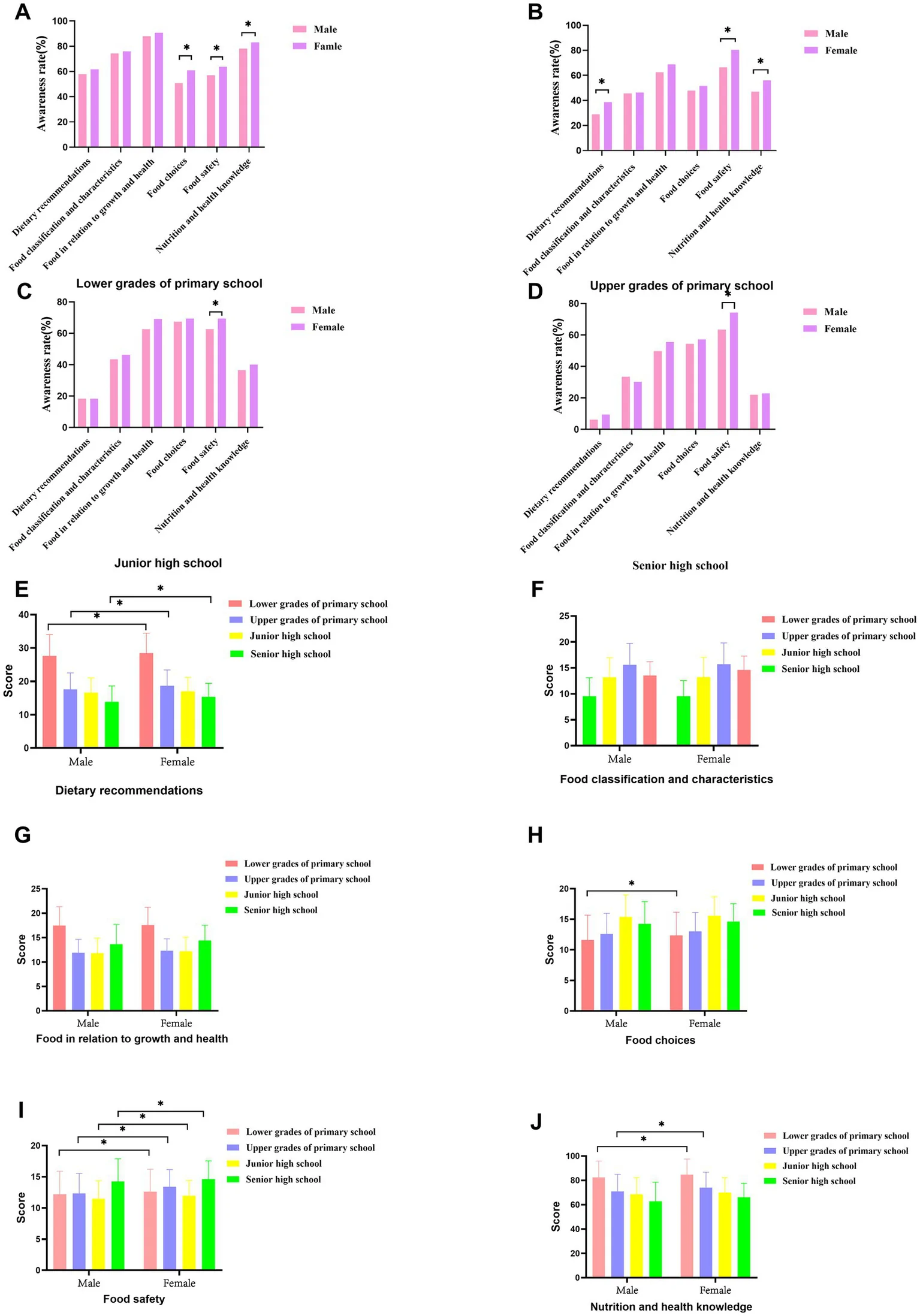
The differences in awareness rates **(A–D)** and scores **(E–J)** of knowledge dimensions among school-age children of different genders.

### 3.4 Knowledge of nutrition and health facts

Regarding the awareness rate of nutrition-related knowledge points, no knowledge point exhibited a correct rate below 25% among lower grades of primary school students. Among upper grades of primary school students, 3 knowledge points had a correct rate below 25%, while the corresponding numbers for junior high school and senior high school students were 7 and 8, respectively. Additionally, 8 knowledge points achieved a correct rate of 75% or higher among lower grades of primary school students; the numbers for upper grades of primary school, junior high school, and senior high school students were 9, 7, and 5, respectively (Table 2).

**Table 2:** Responses to knowledge points.

School stage	Classification by accuracy metrics	Knowledge dimension	Essential points	Accuracy (%)
Lower grades of primary school	50% ≤ accuracy <75%	Dietary recommendation	It is recommended that eggs or other types of eggs should be consumed every day	69.40
			Fruits and vegetables cannot be considered nutritionally interchangeable	67.85
			Dietary combination	54.69
		Food choices	Snack Choices	68.63
		Food safety	Food Storage	72.42
	Accuracy ≥75%	Dietary recommendation	The health benefits of eating breakfast	88.38
			Balanced Diet	87.61
			Beverage recommendations	94.66
			Dietary habits	98.37
		Food classification and characteristics	Cooking methods for soybeans	88.69
			Food identification	86.13
			Food identification	96.51
		Food in relation to growth and health	Sugar-sweetened beverages and oral health	91.48
			Biscuits, cream cakes and obesity	90.70
			The connection between carrots and vision	93.88
			Sun exposure and bone health	76.14
		Food choices	Comparison of fruit juice and whole fruit	86.06
			Comparison of cooking methods	85.13
		Food safety	Food cleanliness	92.10
			Disposal of spoiled food	93.66
Upper grades of primary school	Accuracy <25%	Dietary recommendation	Breakfast habits、Breakfast combinations	3.11
		Food in relation to growth and health	The relationship between oils, salt and health	7.97
			Dark-colored vegetables, whole grains, livestock and poultry meat, and sunlight exposure: implications for health	16.57
	25 ≤ accuracy <50%	Dietary recommendation	Proper hydration	37.61
			Dietary habits	43.75
			Chinese traditional food culture	44.30
			Chinese food guide pagoda — fish, poultry, eggs, and lean meats	38.49
			Chinese food guide pagoda — milks and soybeans	39.92
		Food classification and characteristics	Sun exposure and cutaneous production of vitamin D	43.11
			Physiological functions of nutrients	36.81
			Relationship between food and crops	35.62
		Food choices	Interpreting food labels	29.88
			Foods high in fat and salt	42.07
			Away-from-home food consumption	28.05
		Food safety	Food preparation and dining hygiene recommendations	39.44
	50 ≤ accuracy <75%	Dietary recommendation	Food combination	54.18
			Chinese food guide pagoda —cereals and tubers	62.79
			Chinese food guide pagoda —vegetables and fruits	54.18
		Food classification and characteristics	The nutrient-rich composition of beef	74.98
			The nutrient-rich composition of animal livers	68.13
			The nutrient-rich composition of rice	66.06
			The nutrient-rich composition of fresh jujube	74.74
		Food in relation to growth and health	Sugar-sweetened beverage consumption in relation to health	54.90
			Practices for maintaining a healthy weight	67.89
		Food choices	Mass estimation of common fruit species	61.43
			Food waste prevention strategies	69.24
		Food safety	The handling of food spoilage, wild mushrooms, and wildlife	61.67
	Accuracy ≥75%	Dietary recommendation	Chinese food guide pagoda —oil、salt	78.17
		Food classification and characteristics	Categorization of food items: maize	87.97
			Categorization of food items: banana	97.21
			Categorization of food items: eggplant	90.12
		Food choices	Proper interpretation of food shelf life	78.65
		Food safety	Storage Conditions—raw meat (if not consumed on the same day)	78.33
			Storage Conditions—cut watermelon	85.02
			Storage Conditions—flour	83.75
			Storage Conditions—leftover rice	76.49
Junior high school	Accuracy <25%	Food classification and characteristics	Food sources and processing	14.48
			Compared with refined grains, the characteristics of whole grains	15.08
			Functional correspondence of nutrients	21.42
		Food in relation to growth and health	An accurate understanding of sugar-sweetened beverages	24.16
			Knowledge of snacks	19.45
		Food safety	Disposal methods for near-expiry food, leftovers, spoiled food, and meat frozen for over one year	3.77
			Proper methods of food storage	20.82
	25 ≤ accuracy <50%	Dietary recommendation	A proper understanding of breakfast	37.36
			Chinese food guide pagoda —milks	38.05
			Chinese food guide pagoda —soybeans	30.85
			Chinese food guide pagoda — fish, poultry, eggs, and lean meats	43.02
			Recommended intake of salt for adolescents	32.22
			Recommended intake of added sugars for adolescents	41.30
			Recommended intake of cooking oil for adolescents	25.79
			The characteristics of balanced diet	47.13
			Daily food pairing and combination	35.56
		Food in relation to growth and health	The effects of sodium, fat, and trans fatty acid intake on human health	34.88
			Health benefits of the consumption of various food types	34.19
		Food choices	Proper selection of food items	47.90
	50 ≤ accuracy <75%	Dietary recommendation	Chinese food guide pagoda —vegetables	69.84
			Chinese food guide pagoda —fruit	54.76
			Chinese food guide pagoda —oil	63.24
			Chinese food guide pagoda —salt	58.10
		Food classification and characteristics	The nutrient-rich composition of animal livers	61.44
			The nutrient-rich composition of fresh jujube	67.78
		Food in relation to growth and health	A proper perspective on overweight and obesity	68.98
		Food choices	Use of nutrition labeling	71.38
			Understanding of out-of-home dining	67.69
			Proper practices to avoid food waste	68.55
		Food safety	Food preparation hygiene and the use of communal serving utensils	60.93
			A proper understanding of wildlife, wild mushrooms, and spoiled food	62.47
	Accuracy≥75%	Dietary recommendation	Proper hydration	93.83
			Healthy eating habits	76.69
			Chinese food guide pagoda —cereals and tubers	83.72
		Food classification and characteristics	The nutrient-rich composition of rice	82.60
			The nutrient-rich composition of milk	80.72
		Food choices	Selecting foods based on shelf life	89.37
			Mass estimation of common fruit species	79.78
Senior high school	Accuracy <25%	Dietary recommendation	Chinese food guide pagoda —soybeans	24.46
			Recommended intake of salt for senior high school students	23.59
			Recommended intake of cooking oil for senior high school students	19.68
			Recommendations for a balanced diet	15.46
		Food in relation to growth and health	Knowledge of iron deficiency anemia	5.02
		Food choices	Use of nutrition labeling	21.35
			A proper understanding of snacks	22.31
		Food safety	Disposal methods for near-expiry food, leftovers, spoiled food, and meat frozen for over one year	4.06
	25 ≤ accuracy <50%	Dietary recommendation	A proper understanding of breakfast	43.27
			Chinese food guide pagoda —fruit	40.48
			Chinese food guide pagoda —milk	28.92
			Chinese food guide pagoda — fish, poultry, eggs, and lean meats	27.97
			Recommended intake of added sugars for senior high school students	36.89
			Drinking water and health	49.40
		Food classification and characteristics	Comparison of vitamin C content between freshly squeezed juice and whole fruits	47.01
			Comparison of calcium content in bone broth and milk	34.10
			Dietary sources of different nutrients	29.16
			The function of nutrients	35.14
		Food in relation to growth and health	Criteria for BMI classification	43.35
			Toward a proper understanding of sugar-sweetened beverages	39.28
		Food safety	Proper methods of food storage	27.89
	50 ≤ accuracy <75%	Dietary recommendation	Characteristics of a balanced diet	66.37
			Chinese food guide pagoda —vegetables	56.10
			Chinese food guide pagoda —oil	58.01
			Chinese food guide pagoda —salt	56.25
		Food classification and characteristics	Comparison of dietary fiber content between multigrain rice and white rice	59.68
			A comparative study of sugar content in canned fruit versus fresh fruit	64.54
		Food in relation to growth and health	Body Mass Index calculation formula	51.87
		Food choices	A proper perspective on street food	73.55
			Proper practices for dining out	68.69
			Selection considerations for food productstab3_2	55.06
		Food safety	The cleanliness and hygiene of food and its preparation, as well as the use of serving utensils	65.42
			An accurate understanding of wild animals, mushrooms, and spoiled food	66.61
	Accuracy ≥75%	Dietary recommendation	Healthy eating habits	75.46
			Chinese food guide pagoda —cereals and tubers	76.73
		Food in relation to growth and health	Effective strategies for maintaining a healthy body weight	76.02
		Food choices	Mass estimation of common fruit species	80.00
			Proper practices to avoid food waste	77.05

### 3.5 Analysis of influencing factors

Variables with a *p* < 0.05 in univariate analysis were selected as independent variables, with the awareness of nutrition and health knowledge as the dependent variable for Logistic regression analysis. The results showed that the awareness rate of nutrition and health knowledge was associated with gender, boarding status, awareness of father’s education, and school lunch among lower-grade primary school students; with gender, school breakfast and dinner, awareness of mother’s education and employment, and awareness of father’s employment among upper-grade primary school students; with awareness of mother’s education and employment among junior high school students; and with awareness of mother’s education and employment, boarding status, and school breakfast and dinner among senior high school students (Table 3).

**Table 3:** Logistic regression analysis results of nutrition literacy among school-age children by school stage.

School stage	Independent variable	Category	*β*	SE	*t*	*p*	OR (95% CI)
Lower primary grades	Constant	**—**	1.04	0.20	5.32	0.00	2.83 (1.92 ~ 4.17)
	Gender	Male (Ref: Female)	−0.30	0.13	−2.28	0.03	0.74 (0.57–0.96)
	Boarding student	Yes (Ref: No)	0.79	0.40	1.99	0.05	2.21 (1.00–4.87)
	Father’s education (Ref: Unknown)	Primary school or below	0.26	0.30	0.86	0.39	1.29 (0.71–2.34)
		Junior high school	1.15	0.34	3.36	0.00	3.14 (1.60–6.19)
		Senior high school / vocational school / technical school	1.32	0.41	3.32	0.00	3.75 (1.67–8.44)
		Junior college / vocational college	1.32	0.37	3.60	0.00	3.74 (1.81–7.75)
		Bachelor’s degree	0.94	0.33	2.90	0.01	2.57 (1.35–4.89)
		Master’s degree or above	0.89	0.44	2.01	0.05	2.43 (1.01–5.83)
	School lunch	Not eating (Ref: Eating)	−1.37	0.47	−2.94	0.00	0.25 (0.10–0.64)
Upper primary grades	Constant	**—**	−0.78	0.38	−2.05	0.04	0.46 (0.22 ~ 0.98)
	Gender	Male (Ref: Female)	−0.42	0.14	−3.07	0.00	0.66 (0.50–0.86)
	School breakfast	Not eating (Ref: Eating)	−1.12	0.52	−2.13	0.04	0.33 (0.12–0.93)
	School dinner	Not eating (Ref: Eating)	1.28	0.53	2.37	0.02	3.48 (1.22–9.91)
	Mother’s education (Ref: Unknown)	Primary school or below	−0.19	0.25	−0.74	0.46	0.83 (0.50–1.37)
		Junior high school	0.16	0.25	0.62	0.54	1.17 (0.71–1.93)
		Senior high school / vocational school / technical school	0.18	0.25	0.73	0.47	1.20 (0.73–1.96)
		Junior college / vocational college	0.49	0.39	1.26	0.22	1.63 (0.75–3.56)
		Bachelor’s degree	0.84	0.26	3.19	0.00	2.31 (1.37–3.90)
		Master’s degree or above	0.48	0.36	1.34	0.18	1.62 (0.79–3.30)
	Father’s Employment (Ref: Unknown)	Healthcare institutions	1.01	0.35	2.94	0.00	2.76 (1.39–5.46)
		Other health-related industry	−0.28	0.66	−0.43	0.67	0.76 (0.21–2.78)
		Education-related industry	0.33	0.62	0.52	0.61	1.39 (0.40–4.77)
		Food processing/manufacturing industry	−0.29	0.59	−0.50	0.62	0.75 (0.23–2.39)
		Catering/Food wholesale and retail	−0.32	0.53	−0.61	0.55	0.72 (0.25–2.08)
		Agriculture, forestry, animal husbandry, and fishery	0.44	0.66	0.67	0.50	1.55 (0.42–5.74)
		Others	0.53	0.29	1.81	0.07	1.69 (0.95–3.02)
	Mother’s Employment (Ref: Unknown)	Healthcare institutions	0.51	0.31	1.63	0.11	1.66 (0.90–3.07)
		Other health-related industry	0.91	0.58	1.57	0.12	2.48 (0.79–7.84)
		Education-related industry	0.24	0.44	0.54	0.59	1.27 (0.53–3.00)
		Food processing/manufacturing industry	0.38	0.86	0.44	0.66	1.46 (0.27–8.04)
		Catering/Food wholesale and retail	0.95	0.47	2.03	0.05	2.57 (1.02–6.49)
		Agriculture, forestry, animal husbandry, and fishery	0.15	0.52	0.29	0.77	1.17 (0.41–3.29)
		Others	0.39	0.22	1.73	0.09	1.47 (0.95–2.29)
Junior high school	Constant	—	−1.34	0.25	−5.44	0.00	0.26 (0.16 ~ 0.43)
	Mother’s education (Ref: Unknown)	Primary school or below	0.21	0.27	0.78	0.44	1.23 (0.72–2.09)
		Junior high school	0.62	0.25	2.45	0.02	1.86 (1.13–3.07)
		Senior high school / vocational school / technical school	0.65	0.22	2.94	0.00	1.91 (1.23–2.96)
		Junior college / vocational college	0.56	0.34	1.67	0.10	1.75 (0.90–3.42)
		Bachelor’s degree	0.44	0.31	1.41	0.16	1.56 (0.84–2.91)
		Master’s degree or above	0.25	0.64	0.39	0.70	1.28 (0.36–4.61)
	Mother’s Employment (Ref: Unknown)	Healthcare institutions	0.61	0.46	1.34	0.18	1.85 (0.74–4.59)
		Other health-related industry	1.09	0.57	1.92	0.06	2.98 (0.96–9.19)
		Education-related industry	0.95	0.35	2.69	0.01	2.58 (1.28–5.21)
		Food processing/manufacturing industry	0.37	0.64	0.57	0.57	1.44 (0.40–5.16)
		Catering/Food wholesale and retail	0.55	0.23	2.36	0.02	1.73 (1.09–2.74)
		Agriculture, forestry, animal husbandry, and fishery	0.89	0.28	3.12	0.00	2.42 (1.38–4.26)
		Others	0.39	0.21	1.89	0.06	1.48 (0.98–2.22)
Senior high school	Constant	**—**	−1.02	0.40	−2.56	0.01	0.36 (0.16 ~ 0.80)
	Mother’s education (Ref: Unknown)	Primary school or below	0.31	0.30	1.05	0.30	1.36 (0.76–2.45)
		Junior high school	0.47	0.28	1.69	0.10	1.60 (0.92–2.79)
		Senior high school / vocational school / technical school	0.75	0.31	2.38	0.02	2.11 (1.13–3.94)
		Junior college / vocational college	0.81	0.43	1.86	0.07	2.24 (0.95–5.30)
		Bachelor’s degree	−0.37	0.56	−0.66	0.51	0.69 (0.23–2.09)
		Master’s degree or above	0.41	0.73	0.56	0.58	1.51 (0.35–6.43)
	Mother’s Employment (Ref: Unknown)	Healthcare institutions	0.43	0.61	0.70	0.49	1.53 (0.46–5.12)
		Other health-related industry	0.16	0.61	0.26	0.80	1.17 (0.35–3.96)
		Education-related industry	1.77	0.38	4.61	0.00	5.86 (2.74–12.55)
		Food processing/manufacturing industry	−1.21	0.52	−2.33	0.02	0.30 (0.11–0.84)
		Catering/Food wholesale and retail	0.06	0.33	0.19	0.85	1.06 (0.55–2.05)
		Agriculture, forestry, animal husbandry, and fishery	0.07	0.37	0.19	0.85	1.07 (0.51–2.25)
		Others	0.19	0.26	0.71	0.48	1.21 (0.72–2.03)
	Boarding student	Yes (Ref: No)	−0.79	0.26	−3.03	0.00	0.45 (0.27–0.76)
	School breakfast	Not eating (Ref: Eating)	−0.50	0.23	−2.17	0.03	0.61 (0.39–0.96)
	School dinner	Not eating (Ref: Eating)	−0.77	0.22	−3.52	0.00	0.47 (0.30–0.72)

## 4 Discussion

Based on representative data from the National Survey on Nutritional Knowledge Awareness among School-Aged Children in China, this study described the level of nutritional knowledge among school-aged children in Chongqing, a municipality located in Southwest China. These findings provide baseline data for future nutrition education and promotion efforts targeting this population. The survey revealed that the overall awareness rate of nutritional knowledge among school-aged children in Chongqing was 48.51% during 2023–2024. This rate is higher than the survey results from Southwest China overall (34.2%, covering Chongqing, Sichuan, Guizhou, and Yunnan) (15), Sichuan Province (32.98%) (16), and Hubei Province (27.56%) (17). Nevertheless, over half of the school-aged children still failed to meet the threshold for adequate awareness, indicating substantial room for improvement in nutritional knowledge among school-aged children in Chongqing.

In terms of distribution across educational stages, the awareness rates of nutrition and health knowledge were 80.56% for lower-grade primary school students, 51.39% for upper-grade primary school students, 38.30% for junior high school students, and 22.42% for senior high school students. The underlying reasons may be attributed to the implementation of widespread food education programs in China starting in 2023 (18), which aim to guide students in developing scientific dietary habits. Younger children benefit from a greater number of food education courses and activities. In contrast, as older students face increasing academic pressures, the proportion of nutrition education courses and activities gradually decreases (19). Furthermore, senior high school students encounter more independent dietary choices, with their primary dining venues being school canteens or off-campus restaurants (20, 21). Consequently, they lack the family-based nutritional education in areas such as dietary recommendations, food safety, and healthy food selection that is more prevalent among students in other educational stages (22–24).

In terms of awareness across specific dimensions, the “dietary recommendations” dimension remained at a relatively low level across all educational stages, which was particularly pronounced among senior high school students (7.81%). Currently, the intake of whole grains and legumes, fresh vegetables, fruits, dairy products, soybeans, and nuts among Chinese children aged 6–17 years is below the recommended levels. Furthermore, meat consumption is disproportionately derived from livestock (red meat), accounting for approximately 80% (25, 26). These findings are consistent with the unbalanced dietary patterns observed among school-aged children in China, indicating that knowledge of the “Chinese Food Guide Pagoda” remains the most significant gap in nutritional knowledge for this population. Therefore, further efforts should be made to strengthen the dissemination of fundamental nutritional theories.

Conversely, students across all educational stages demonstrated relatively higher awareness rates in the dimensions of “food, growth and development, and health” as well as “food safety.” A previous survey on nutritional knowledge awareness among adult residents in Chongqing similarly reported high accuracy rates in areas related to nutrition and disease, food characteristics, and food safety (27). This trend may be attributed to Chongqing’s continuous implementation and deepening of strategies to popularize nutritional knowledge. These efforts have effectively enhanced the nutritional literacy of both the general public and school-aged children, particularly through comprehensive education and outreach on topics closely related to physical health, such as the impact of food on children’s growth and development, healthy food choices, and food safety.

This study conducted an in-depth analysis of the specific knowledge points covered in each version of the questionnaire. Based on the correct response rates, these knowledge points were systematically reviewed and categorized to assess the deficiencies in nutritional knowledge among children across different educational stages and to identify priority areas for future educational interventions. Across all educational stages, knowledge points with a correct response rate below 25% included breakfast composition, the relationship between the intake of oil, salt, and sugar and health, the health implications of dark-colored vegetables, coarse grains, meat, and soy products, the functions of nutrients, the recognition and selection of snacks and sugar-sweetened beverages, the application of nutrition labels, and the proper handling and storage of food. These areas primarily involve applied nutritional knowledge, indicating that although the school-aged children in this study possess a certain level of theoretical understanding of nutrition and health, they exhibit significant deficiencies in practical judgment and application in daily life, making it difficult for them to make scientifically sound choices. Therefore, it is recommended that nutrition and health education extend from “knowledge” to “practice.” Food education curricula tailored to the characteristics of students at different educational stages should be developed (28–31). Approaches such as visual teaching, simulated shopping, and nutrition laboratory classes can be utilized to enhance students’ nutritional judgment and behavioral execution skills in real-world scenarios.

Logistic regression analysis indicated that gender, school meals, boarding status, and awareness of parents’ education and employment type were associated with the level of nutrition and health knowledge among children across different educational stages. The awareness rate among girls was significantly higher than that among boys during the primary school stage, but this gender difference disappeared in junior and senior high school. This may be attributed to girls’ earlier cognitive development prior to puberty, which potentially gives them an advantage in mastering nutrition and health knowledge (24, 32). The association between eating breakfast, lunch, or dinner at school and the awareness rate of nutrition and health knowledge varied across different educational stages. Overall, children who dined at school exhibited higher awareness rates. This may be due to the implementation of nutritional health management in school canteens in China in 2021 (33), which mandates the dissemination of nutrition and health knowledge to students. Additionally, studies have shown that combining the school meal environment with nutrition education can effectively improve children’s nutrition and health knowledge levels (15). Boarding students in lower-grade primary school had a higher awareness rate (*OR* = 2.21), whereas boarding students in senior high school had a lower rate (*OR* = 0.45). This further suggests that senior high school students may face greater academic pressure, leading to a gradual reduction in the proportion of nutrition health education courses and activities (19). This indicates that the senior high school stage is a weak link in nutrition education, highlighting an urgent need for targeted interventions.

This study showed that, compared with children who were unaware of their parents’ education, those who knew their fathers had a higher educational level exhibited a higher awareness rate of nutrition and health knowledge in lower-grade primary school. Knowing that the mother had a bachelor’s degree was associated with a higher awareness rate in upper-grade primary school children, while knowing that the mother had a senior high school/vocational/technical school education was associated with a higher awareness rate in junior and senior high school students. This suggests that parental educational level is associated with the awareness of nutrition and health knowledge among children across different educational stages. Compared with children who were unaware of their parents’ employments, those who knew their fathers worked in healthcare institutions had a higher awareness rate in upper-grade primary school. Knowing that mothers worked in education-related industries, catering/food wholesale and retail, or agriculture/forestry/animal husbandry/fisheries was associated with a higher awareness rate in junior high school students. Knowing that mothers worked in education-related industries was associated with a higher awareness rate in senior high school students, whereas knowing that mothers worked in food processing/manufacturing industry was associated with a lower awareness rate in this group. Parental education and employment influence the home food environment, which indirectly affects children’s nutrition and health knowledge levels (34). Meanwhile, as children grow and develop, home nutrition education and the food environment also change, thereby altering their impact on children’s knowledge levels (20, 35). Future research could conduct targeted studies on nutrition education and the food environment for children and their families across different educational stages to further clarify these influencing factors.

This study has several limitations. First, the reliability (*Cronbach’s α*) of the questionnaire for lower primary school students was 0.54, which may not accurately reflect the true nutritional knowledge levels in this specific group. Second, Although each version demonstrated acceptable reliability and construct validity, measurement equivalence or score linking across the four versions was not established. The observed differences in knowledge levels across stages may reflect variations in item difficulty or content coverage rather than true developmental trajectories. Third, this study adopted a cross-sectional survey design, which only allows for the analysis of factors associated with children’s awareness of nutrition knowledge, but cannot establish causal relationships between variables. Fourth, due to the unavailability of detailed population demographic information, we were unable to calculate sampling or post-stratification weights, and weights were set to ‘1’ in the complex sampling analysis. This may affect the generalizability of our estimates to the broader target population. Therefore, the findings should be interpreted with caution regarding their representativeness. Fifth, the questionnaire focused on the assessment of knowledge levels and only included general sociodemographic variables. It did not cover key factors that may affect awareness rates, such as whether children had received nutrition education, or the specific channels and methods of such education. Future targeted studies are recommended to further explore these aspects.

## 5 Conclusion

In conclusion, there is still substantial room for improving the level of nutritional knowledge among school-aged children in Chongqing. Significant knowledge gaps exist across different educational stages and dimensions, particularly regarding dietary recommendations, breakfast composition, the relationship between recommended intakes of oil, salt, and sugar and health, the recognition and selection of snacks and sugar-sweetened beverages, and the proper handling and storage of food. It is recommended to develop tiered and progressive nutrition education curricula tailored to the characteristics of different educational stages and knowledge dimensions. Efforts should prioritize the dissemination of core dietary guidelines and applied nutritional knowledge. Furthermore, it is essential to leverage the synergistic roles of families, schools, and society to formulate precise intervention strategies, thereby enhancing the nutritional literacy of school-aged children.

## Data Availability

The raw data supporting the conclusions of this article will be made available by the authors, without undue reservation, subject to strict verification of usage conditions. The datasets generated during the current study are available for data synthesis and review. Requests to access the datasets should be directed to PF, 1065628960@qq.com.
